# Effects of varying inhaled oxygen concentrations on lung function in older adult patients undergoing laparoscopic gastrointestinal surgery under general anesthesia: protocol of a prospective multicenter clinical study in China

**DOI:** 10.1186/s13063-026-09471-3

**Published:** 2026-01-23

**Authors:** Tianhao Zhang, Yang An, Shiling Zhao, Fei Han, Lining Huang, Li Wang, Jianbo Wu, Qian Lei, Kun Wang, Jianlin Shao, Yun Wang, Yong Luan, Wei Feng, Jiannan Song, Zeqing Huang, Chaoran Wu, Yongshan Nan, Bing Tang, Xijia Sun, Wenfei Tan

**Affiliations:** 1 Department of Anesthesiology, The First Hospital of China Medical University, Shenyang, China; 2 Department of Anesthesiology, Dalian Third People’s Hospital, Dalian, China; 3 Department of Anesthesiology, Harbin Medical University Cancer Hospital, Harbin, China; 4 Department of Anesthesiology, The Second Hospital of Hebei Medical University, Shijiazhuang, China; 5 Department of Anesthesiology, The First Hospital of Hebei Medical University, Shijiazhuang, China; 6 Department of Anesthesiology, The First Affiliated Hospital of Shandong First Medical University and Shandong Provincial Qianfoshan Hospital, Jinan, China; 7 Department of Anesthesiology, Sichuan Provincial People’s Hospital, Chengdu, China; 8 Department of Anesthesiology, The First Affiliated Hospital of Harbin Medical University, Harbin, China; 9 Department of Anesthesiology, The First Affiliated Hospital of Kunming Medical University, Kunming, China; 10 Department of Anesthesiology, Beijing Friendship Hospital, Capital Medical University, Beijing, China; 11 Department of Anesthesiology, The First Affiliated Hospital of Dalian Medical University, Dalian, China; 12 Department of Anesthesiology, The Affiliated Hospital of Qingdao University, Qingdao, China; 13 Department of Anesthesiology, Chifeng Municipal Hospital, Chifeng, China; 14 Department of Anesthesiology, Liaoning Cancer Hospital & Institute, Shenyang, China; 15 Department of Anesthesiology, Shenzhen People’s Hospital, Shenzhen, China; 16 Department of Anesthesiology, Yanbian University Hospital (Yanbian Hospital), Yanji, China

**Keywords:** Postoperative pulmonary complications, Laparoscopic gastrointestinal surgery, Fraction of inspired oxygen, Absorption atelectasis, Older adult patients

## Abstract

**Background:**

Postoperative pulmonary complications (PPCs) are severe and are of particular concern in older adult patients undergoing laparoscopic gastrointestinal surgery. Both 40% and 80% fraction of inspired oxygen (FiO_2_) are commonly used for anesthesia. Presently, whether 40% FiO_2_ can increase the oxygenation index of patients 48 h postoperatively and reduce PPCs remains controversial. Moreover, no clear consensus exists for older adult patients. Therefore, this study aims to compare the effects of low FiO_2_ (40%) and high FiO_2_ (80%) levels on postoperative pulmonary function in older adult study participants undergoing laparoscopic gastrointestinal surgery.

**Methods:**

This multicenter, prospective, parallel-cohort, randomized controlled clinical trial will include 1098 older adult participants aged ≥ 65 years old undergoing laparoscopic gastrointestinal surgery, from 16 clinical trial sites across China. Participants will be randomized, as per a 1:1 ratio to two cohorts, the “L” and “H” cohorts, to receive low FiO_2_ (40%) and high FiO_2_ (80%) levels, respectively. The primary outcome measure is the 48-h postoperative oxygenation index between the two cohorts. The secondary outcome measures include the other blood gas analysis results, PPCs within 7 days, and 30-day mortality rate.

**Discussion:**

This study of elderly patients undergoing laparoscopic gastrointestinal surgery with different intraoperative oxygen concentrations at high risk for pulmonary complications. All subjects were followed up for up to 30 days for pulmonary function, postoperative complications, etc. Randomization was performed separately at 16 sites.

**Trial registration:**

ClinicalTrials.gov NCT06359106. Registered and posted on April 11, 2024.

**Supplementary Information:**

The online version contains supplementary material available at 10.1186/s13063-026-09471-3.

## Administrative information

**Table:** 

Title {1}	Effects of varying inhaled oxygen concentrations on lung function in older adult patients undergoing laparoscopic gastrointestinal surgery under general anesthesia: protocol of a prospective multicenter clinical study in China
Trial registration {2a and 2b}	ClinicalTrials. gov (NCT06359106)
Protocol version {3}	2024.6.24 V3.0
Funding {4}	The work was supported by the National Natural Science Foundation of China (Grant No. 82171187) and Wu Jieping Medical Foundation (Project No. 320.6750.2024-05.2024-14)
Author details {5a}	Tianhao Zhang Yang An Shiling Zhao Fei Han Lining Huang Li Wang Jianbo Wu Qian Lei Kun Wang Jianlin Shao Yun Wang Yong Luan Wei Feng Jiannan Song Zeqing Huang Chaoran Wu Yongshan Nan Bing Tang Xijia Sun Wenfei Tan Correspondence to Dr. Wenfei Tan
Name and contact information for the trial sponsor {5b}	Wenfei Tan; wftan@cmu.edu.cn
Role of sponsor {5c}	The sponsor is responsible for coordinating the operations across research centers during the trial implementation phase to ensure the study progresses in accordance with the protocol. During the analysis phase, the sponsor participates in the development and review of the statistical analysis plan, as well as the interpretation and discussion of the analytical results. In the reporting phase, the sponsor oversees the preparation and submission of the final report. The sponsor retains ultimate authority over all aforementioned activities

## Introduction

### Background and rationale {6a}

The World Health Organization (WHO) and United States Center for Disease Control recommend the use of 80% oxygen in the perioperative period, to reduce surgical site infections after general anesthesia [1, 2]. Moreover, clinical anesthesiologists use pure oxygen during the induction and awakening phases of general anesthesia to prevent hypoxemia, particularly in patients with a risk of difficult airways and critical illnesses [3, 4].

However, controversy exists regarding the safety of the perioperative use of a high fraction of inspired oxygen (FiO_2_). With a difficult airway, 100% FiO_2_ is usually administered during a short period, due to the risk of prolonged apnea. In particular, this short inhalation of pure oxygen causes absorption atelectasis in 90% of patients [5, 6], which may be present postoperatively [7, 8]. Absorption atelectasis results in reduced lung compliance, oxygen damage, and impaired oxygen delivery and lung function. The results of a meta-analysis have revealed an association between decreased oxygen levels and parameters and increased atelectasis severity, after high FiO_2_ during anesthesia [9, 10].

Postoperative atelectasis is an important risk factor for postoperative pulmonary complications (PPCs), such as pneumonia, resulting in increased postoperative morbidity and mortality rates and prolonged hospital stays [11–13]. Therefore, PPCs are severe and are of particular concern in older adult patients undergoing laparoscopic gastrointestinal surgery.

A study involving 73,992 participants found that a high FiO_2_ increases PPCs and the 30-day postoperative mortality rate [14].

Moreover, further studies have uncovered that in acute and severe cases, the administration of high concentrations of FiO_2_ does not improve important clinical outcomes and results in an increased mortality rate [15].

Conversely, the findings of a few meta-analyses have revealed that the use of high FiO_2_ during general anesthesia does not increase postoperative mortality, intensive care unit (ICU) occupancy, pneumonia, and atelectasis [16, 17]. Furthermore, the results of a controlled intervention trial comprising 5749 patients have revealed the absence of an effect of high FiO_2_ levels on 30-day mortality rates or wound healing [18, 19].

Excessive oxygen directly results in toxic effects involving the lung [20]. Furthermore, excessive oxygen induces vasoconstriction, inflammatory responses, and oxidative stress, impacting on the pulmonary, cardiovascular, and nervous systems [21, 22]. Moreover, a high FiO_2_ administered during surgery lacks evidence regarding the benefits thereof for postoperative infection [23].

PPCs frequently occur post-lung resection and have a considerable impact on postoperative morbidity and mortality. The prevalence of PPCs post-lung resection, which ranges from 14 to 59%, is notably higher than that of other major surgeries that do not require single-lung ventilation [24–27].

Thus, employing a ventilation strategy that protects the lungs can decrease the incidence of PPCs [28, 29]. Due to the inconsistency of evidence, the 2019 International Expert Development Consensus on Lung-Protective Ventilation has not recommended any specific mode of ventilation [29].

Pressure-controlled ventilation (PCV) results in better oxygen and inspiratory pressure supply, an improved lung compliance, and a reduced release of inflammatory mediators, in patients undergoing thoracic and abdominal surgery [30–43].

However, an analysis involving 109,360 patients revealed a notable increase in PPCs in the PCV group than that in the volume-controlled ventilation (VCV) group [38]. As has been recommended for intraoperative use, for reducing airway pressure and improving oxygenation, the PCV-volume guaranteed ventilation (VG) mode is preferred, based on the characteristics of PCV and VCV [37–39]. Nonetheless, no conclusive evidence exists indicating that any mode of ventilation can reduce the incidence of PPCs.

Current evidence supports low FiO_2_ for positive clinical outcomes; however, the latest guidelines recommend an intraoperative FiO_2_ with a lower limit of 40% [43]. The WHO Guidelines Development Group suggests that the FiO_2_ in patients undergoing endotracheal intubation under general anesthesia should be 80%, to reduce the incidence of postoperative atelectasis [44].

Moreover, FiO_2_ is generally set at 30% to 100% in clinical practice. Recommendations include adding air to the inhaled gas and adjusting the FiO_2_ from 30 to 40%.

Therefore, both 40% and 80% FiO_2_ are commonly used for anesthesia. Presently, whether 40% FiO_2_ can increase the oxygenation index of patients 48 h postoperatively and reduce PPCs remains controversial. Moreover, at present, no consensus exists regarding the best intraoperative FiO_2_ to be used for lung function under anesthesia, whether spontaneous breathing or endotracheal intubation is employed. No clear consensus exists for older adult patients.

This multicenter, prospective, parallel-cohort, randomized controlled clinical study aims to compare the effects of low FiO2 (40%) and high FiO2 (80%) levels on postoperative pulmonary function in older adult participants undergoing laparoscopic gastrointestinal surgery.

### Objectives {7}

#### Primary objective

To investigate whether varying levels of intraoperative FiO_2_ affect the 48-h postoperative oxygenation index in older adult participants who have undergone laparoscopic gastrointestinal surgery.

#### Secondary objectives

1. To compare the results of blood gas analyses including PH value and PaCO_2_ lactic acid between the two cohorts, within 48 h postoperatively.
2. To compare the time of oxygen inhalation, the inspired oxygen concentration, and the rate of oxygen uptake
3. To compare the 7-day pulmonary and 30-day mortality rate between the two cohorts

#### Trial design {8}

**Fig. 1:**
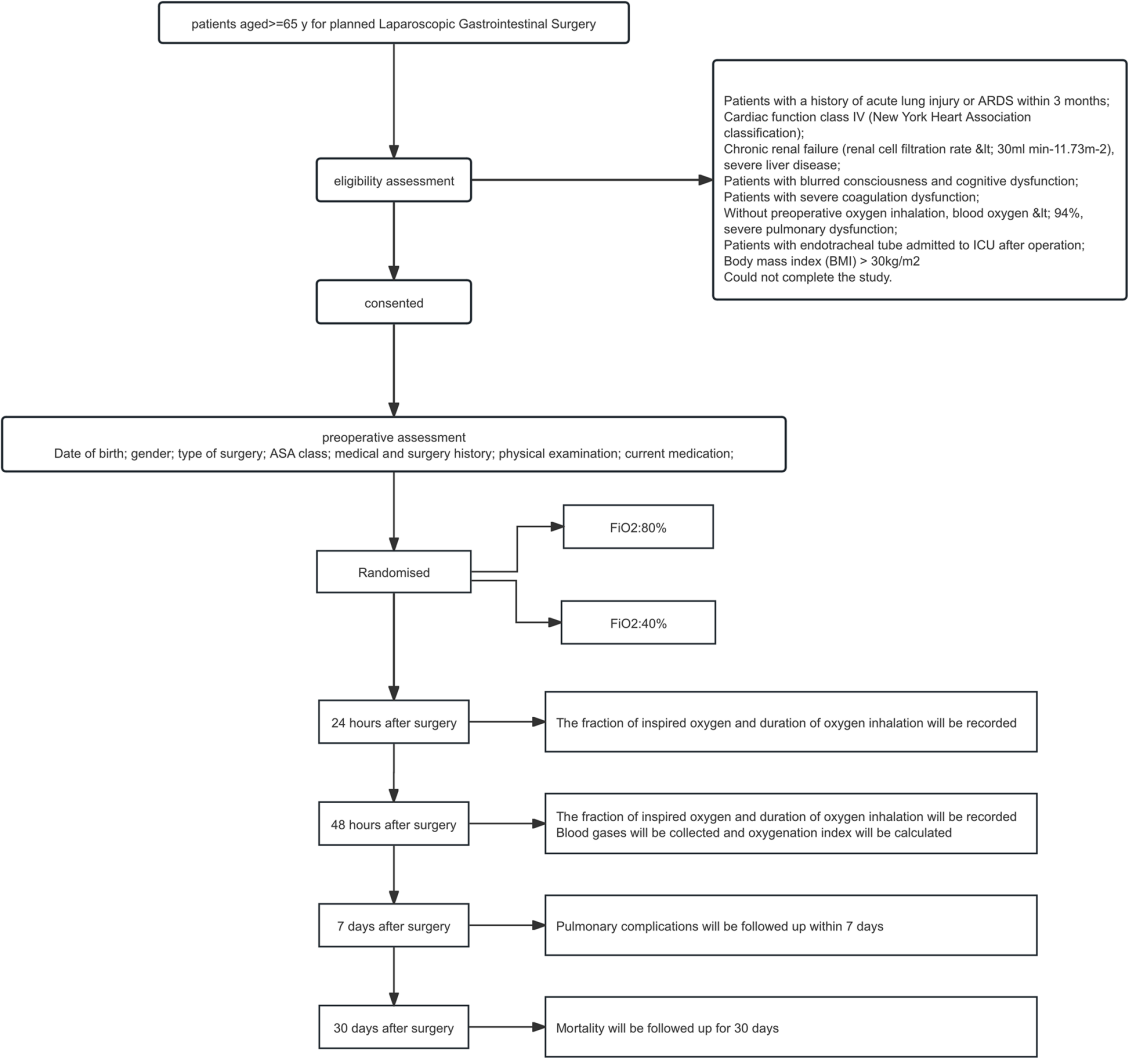
Trial flowchart

The planned study is a multicenter, prospective, parallel-cohort, randomized controlled clinical trial, and the start date is 2 July 2024, with an estimated end date of 31 December 31, 2025. Figure 1 illustrates the trial design.

## Methods: participants, interventions, and outcomes

### Study setting {9}

This study will be performed at 16 clinical trial sites across various regions of the Chinese Mainland. The 16 sites include the following:

- Department of Anesthesiology, The First Hospital of China Medical University, Shenyang, China
- Department of Anesthesiology, Dalian Third People’s Hospital, Dalian, China
- Department of Anesthesiology, Harbin Medical University Cancer Hospital, Harbin, China
- Department of Anesthesiology, The First Affiliated Hospital of Dalian Medical University, Dalian, China
- Department of Anesthesiology, The Affiliated Hospital of Qingdao University, Qingdao, China
- Department of Anesthesiology, The Second Hospital of Hebei Medical University, Shijiazhuang, China
- Department of Anesthesiology, The First Hospital of Hebei Medical University, Shijiazhuang, China
- Department of Anesthesiology, Chifeng Municipal Hospital, Chifeng, China
- Department of Anesthesiology, The First Affiliated Hospital of Shandong First Medical University & Shandong Provincial Qianfoshan Hospital, Jinan, China
- Department of Anesthesiology, Sichuan Provincial People’s Hospital, Chengdu, China
- Department of Anesthesiology, The First Affiliated Hospital of Harbin Medical University, Harbin, China
- Department of Anesthesiology, Liaoning Cancer Hospital & Institute, Shenyang, China
- Department of Anesthesiology, The First Affiliated Hospital of Kunming Medical University, Kunming, China
- Department of Anesthesiology, Shenzhen People’s Hospital, Shenzhen, China
- Department of Anesthesiology, Beijing Friendship Hospital, Capital Medical University, Beijing, China
- Department of Anesthesiology, Yanbian University Hospital (Yanbian Hospital), Yanji, China

### Eligibility criteria {10}

The inclusion criteria are as follows:

1. Participants aged ≥ 65 years old
2. Participants with Grades I–III, as per the American Society of Anesthesiologists (ASA) Physical Status Classification System.
3. Participants without a previous history of drug allergies or abnormal reactions to anesthesia
4. Participants with an operative time > 2 h
5. Participants undergoing laparoscopic gastrointestinal surgery
6. Participants with a preoperative oxygen saturation not < 94%
7. Participants for whom extubation is planned in the operating room

The exclusion criteria are as follows:

1. Patients who have experienced acute lung injury or acute respiratory distress syndrome in the past 3 months
2. Patients with a cardiac function class IV, as per the New York Heart Association classification
3. Patients with chronic renal failure, as evidenced by an estimated glomerular filtration rate of 30 mL/min/1.73 m^2^
4. Patients with severe hepatic disease
5. Patients with blurred consciousness or cognitive dysfunction
6. Patients with severe coagulation dysfunction
7. Patients without a measurement of preoperative oxygen inhalation
8. Patients with blood oxygen level < 94%
9. Patients with severe pulmonary dysfunction
10. Patients intubated with an endotracheal tube who are admitted to ICU, postoperatively
11. Patients with a body mass index > 30 kg/m^2^

The rejection criteria are as follows:

1. Participants who could not complete the study
2. Participants with life-threatening cardiovascular and cerebrovascular events occurring intraoperatively
3. The operation involves the thoracic cavity or is converted to open surgery.
4. The surgical procedure lasts > 8 h.
5. The amount of bleeding is > 800 ml.

The intervention will be handled by an experienced anesthesiologist. Prior to study initiation, researchers will receive training on the study protocol, according to the principles of GCP for clinical trials.

### Who will take informed consent? {26a}

A trained research team will recruit participants and use standard randomization procedures, to assign them to the study population on the preoperative day.

### Additional consent provisions for collection and use of participant data and biological specimens {26b}

Not applicable. The preoperative signed informed consent form thoroughly informed the patient about the planned arterial blood sampling and obtained their consent.

## Interventions

### Explanation for the choice of comparators {6b}

Both 40% and 80% FiO2 are widely used in clinical anesthesia. However, there is currently no definitive conclusion regarding their respective benefits in reducing postoperative pulmonary complications in elderly patients. The study will comprise two cohorts, the “L” and “H” cohorts, to receive low FiO2 (40%) and high FiO2 (80%), respectively. Interventions will be performed as per the clinical practices established at either the national or local levels in China. The trial flowchart is shown in Fig. 1.

### Intervention description {11a}

Preoperatively, the participants in both cohorts will fast for 8 h and not use any sedatives or analgesics. On arrival at the ward/operating room, the data will be verified, and standard monitoring of the systolic and diastolic blood pressure, mean arterial pressure, pulse rate, electrocardiogram, blood oxygen saturation, entropy index, or bispectral index (BIS) will be conducted. Simultaneously, venous access will be obtained and maintained at a rate of 10–20 mL/kg/h. The total fluid volume will not exceed 50 mL/kg, to ensure intraoperative hemodynamic stability.

Intraoperatively, the same protocol will be used for anesthesia induction: etomidate 0.3 mg/kg or propofol 1.5–2 mg/kg, sufentanil 0.3–0.5 μg/kg, cisatracurium besylate 0.2 mg/kg, or rocuronium bromide 1 mg/kg, respectively. Anesthesia methods, including combined intravenous and inhalation anesthesia, total intravenous anesthesia, inhalation anesthesia, and reasonable use of vasoactive drugs, can be used intraoperatively.

The ventilator will be connected to the participant after a successful tracheal intubation. All participants will be ventilated using lung-protective ventilation, which includes VCV, PCV, and PCV-VG.

The tidal volume will be set at 6–8 mL/kg, which has been calculated, based on the ideal body weight, using the following formulae:

- For males: 50 + 0.91 × [height (cm)—152.4].
- For females: 45.5 + 0.91 × [height (cm)—152.4].

Moreover, the positive end-expiratory pressure (PEEP) and inspiratory/expiratory ratio will be maintained at 6–8 cm H2O and 1:2, respectively, with regular lung recruitment maneuvers. The ventilation rate will be adjusted, according to the intraoperative end-expiratory carbon dioxide, which will be maintained at 35–50 mm Hg.

The depth of anesthesia will continuously be adjusted, according to the entropy index, BIS value, or minimum alveolar concentration (MAC) value of the end-expiratory anesthetic gas. The entropy index and BIS value or MAC value of the end-expiratory anesthetic gas will be maintained at 40–60 or 0.7–2.6, respectively.

Intraoperative and perioperative information, adverse events (AEs), and serious adverse events (SAEs) will be recorded. Perioperative data includes the duration of the anesthesia and surgery, use of different types of anesthetics, volumes of fluids administered intraoperatively, and administration of postoperative analgesics and anticholinergic drugs.

The endotracheal tube will be removed in the operating room. If an endotracheal tube is inserted in the PACU, the ventilator parameters in the PACU should consistently be set intraoperatively, and the endotracheal tube should be removed after rapid recovery of spontaneous breathing. Both cohorts will be given Venturi masks for oxygen inhalation post-extubation, and the FiO2 will be set at 24%–40%. The initial setting will be at 24%. If clinical requirements are not met, the FiO2 will be increased to 40%, as needed. The FiO2 and duration of oxygen inhalation will be recorded.

After returning to the ward, the participants will decide whether to receive supplemental oxygen, based on their present condition. If oxygen is required, a Venturi mask will be used. The total time and concentration of oxygen inhalation will be recorded. Each site can use multimodal analgesia, as per the current condition.

During the postoperative monitoring period, evaluations will be conducted, and data will be documented for both the primary and secondary outcome measures.

### Criteria for discontinuing or modifying allocated interventions {11b}

The FiO_2_ will be adjusted to 40% and 80% in the L and H cohorts, respectively. Should the intraoperative oxygen saturation of the participants in the L cohort decline to < 94%, while the PEEP remains at 30 cm H_2_O, at a duration of ≥ 30 s, without an improvement in the oxygen saturation, the oxygen concentration will be increased to 80%.

### Withdrawal from study

Participants will be withdrawn from the study, if any of the following circumstances arise:

1. The participant has voluntarily withdrawn consent from the study.
2. Continuing in the trial will may be harmful to the health of the participant.
3. The trial records cannot be completed, and the research investigator regards the participant as no longer able being to continue in the trial.
4. AEs related to the trial intervention occur.
5. Factors posing a risk are considered by the Trial or Advisory Committee.
6. The rationale for a participant’s withdrawal from the study will be documented in the “Withdrawal/Change of Status” table.

### Strategies to improve adherence to interventions {11c}

We plan to implement the following measures to enhance patient compliance:

1. Thoroughly explain to patients that both oxygen concentrations are clinically established and medically acceptable methods
2. Arrange for arterial blood sampling postoperatively to be performed by experienced anesthesiologists and coordinate with the ward staff to incorporate it into routine postoperative testing

### Relevant concomitant care permitted or prohibited during the trial {11d}

N/A. There are no restrictions on relevant concomitant care.

### Provisions for posttrial care {30}

N/A. Both oxygen concentrations are clinically safe for completing surgical procedures and are not expected to cause harm to participants. Therefore, no compensation will be provided.

### Outcomes {12}

#### Primary outcome

The primary outcome measure is the comparison of the 48-h postoperative oxygenation index between the two cohorts. This will be derived from arterial blood gas sampling and analysis, in addition to the FiO_2_ 48th h postoperatively.

#### Secondary outcomes

1. The results of blood gas analyses including PH value and PaCO_2_ lactic acid between the two cohorts, within 48 h postoperatively
2. The time of oxygen inhalation, the inspired oxygen concentration, and the rate of oxygen uptake
3. The 7-day pulmonary and 30-day mortality rate between the two cohorts

### Participant timeline {13}

**Fig. 2:**
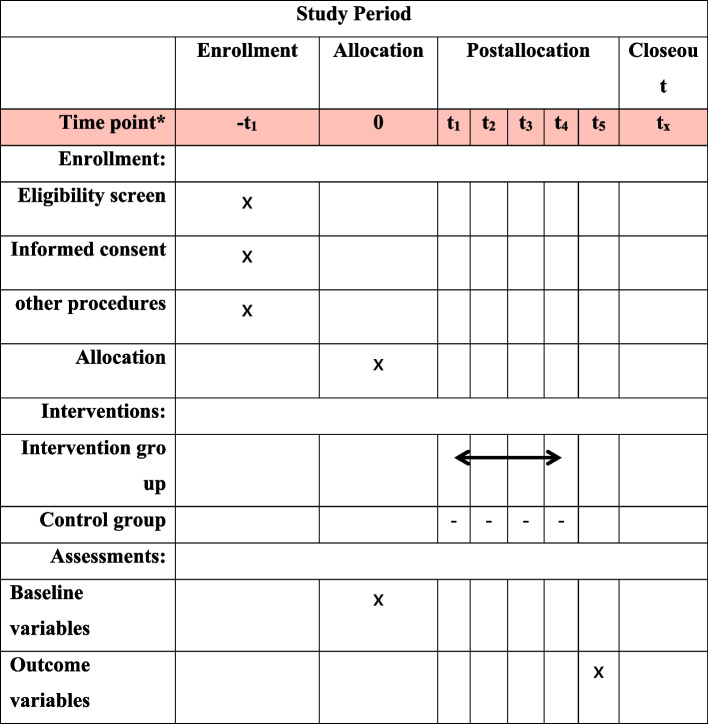
Enrollment, interventions, assessments, and visits for participants

The enrollment, interventions, assessments, and visits for participants are shown in Fig. 2.

### Sample size {14}

This randomized controlled trial aimed to examine the effect of varying intraoperative oxygen concentrations on PPCs. This study is a superiority trial in which the optimal effect threshold has been set. Based on a preliminary experiment, 10 samples were collected each group, and the values of oxygenation index were expressed as mean ± standard deviation: 384 ± 85 mmHg for 40% group and 340 ± 60 mmHg for 80% group. This study was designed as a superiority trial, with a clinically significant difference defined as an increase in oxygenation index by at least 30 (10% of 300 acute lung injure oxygenation index) in favor of 40% group compared to 80% group. The pooled standard deviation was estimated to be 74 mmHg. The sample size required for each group to achieve a significant difference, with a one-sided *α* = 0.025 and *β* = 0.20. The minimum sample size required for each group was approximately 439, with a total of 1098 samples needed for both groups, considering a dropout rate of 20%.

### Recruitment {15}

Participants will be enrolled through being scheduled for surgical procedures, such as laparoscopic gastrointestinal surgery for cancer. For prospective recruitment, an information sheet will be provided regarding the trial objectives, workflow, and randomization procedures. Prior to the procedure, the participant will provide written informed consent. In cases where the participant is unable to do so, a legally authorized representative or caregiver will be contacted to provide consent on behalf of the participant, in accordance with the GCP guidelines.

Preoperative visits will be conducted; and the inclusion and exclusion criteria will be strictly employed. Participants who meet all the eligibility criteria and provide written informed consent will be eligible for enrollment. A trained research team will recruit participants and use standard randomization procedures, to assign them to the study population on the preoperative day. The anesthesiologist will only be notified of this assignment the day after the participant is admitted to the ward.

## Assignment of interventions: allocation

### Sequence generation {16a}

Post-initiation of the study, each clinical trial site will continuously screen eligible participants and enroll them in the trial. Participants will be randomly assigned. The study employed block randomization with center stratification, where each center competed for inclusion. The electronic data capture (EDC) system generated the randomization. The participants will be assigned to two cohorts, as per a 1:1 ratio, on the preoperative day. To balance the variables, we will stratify the participants according to the following factors: age, surgical position, area of the clinical trial site, operative time, and the anesthetic drug administration and method.

### Concealment mechanism {16b}

Using a block randomization EDC system for allocation concealment.

### Implementation {16c}

A trained research team will recruit participants and use standard randomization procedures, to assign them to the study population on the preoperative day. The anesthesiologist will only be notified of this assignment the day after the participant is admitted to the ward.

## Assignment of interventions: blinding

### Who will be blinded {17a}

Due to the nature of the study, anesthesiologists will not be blinded to the intervention protocol. Trial participants, outcome assessors, and data analysts will be blinded. To reduce potential bias, data collectors will be divided into two different teams: (1) anesthesiologists on duty in the operating room on the same day, in addition to postanesthetic care unit (PACU) staff, who will participate in the calculation and evaluation of the results, and (2) blinded principal investigator (PI) and co-investigator, statistician, and data collection and outcome evaluation personnel, which includes those who will provide care and evaluate participants in the ward pre- and postoperatively.

### Procedure for unblinding if needed {17b}

Emergency unblinding will be permitted only when knowledge of the assigned intervention is essential for the clinical management of a serious adverse event.

## Data collection and management

### Plans for assessment and collection of outcomes {18a}

Each site will administer anesthesia, according to their institutional guidelines. Medication type, dosing, and additional analgesics administered, as per each site’s anesthesia protocol, will be documented, in detail, in the case report form (CRF). A qualified and experienced anesthesiologist will be required to complete the CRF.

Prior to study initiation, researchers will receive training on the study protocol, according to the principles of GCP for clinical trials.

### Plans to promote participant retention and complete follow-up {18b}

Data collection and outcome evaluation personnel will be conducted as in-person visits with participants from the preoperative day to 7 days postoperatively. Moreover, these personnel will follow up with participants or their guardians, telephonically, for 30 days postoperatively to gather data. The ARISCAT risk score will be used by the data collection and outcome evaluation personnel to evaluate the risk of PPCs.

### Data management {19}

The data will be managed and analyzed according to the GCP guidelines and handled with strict confidentiality. Trained professionals will enter all trial data into an electronic CRF (eCRF). The eCRF should be completed promptly after data collection, and any missing data should be appropriately explained. A copy of the clinical trial sites’ data will be retained. A data inspector will verify the accuracy and completeness thereof, before sending the eCRF to the trial scheduling center.

Clinical quality inspectors will monitor the data at each center, and the data will be checked by means of electronic CRFS at a frequency of every 30 cases.

### Confidentiality {27}

The collected data will be processed anonymously and stored securely for the exclusive purposes of the researchers and supervisors. Data will be maintained for up to 5 years to support the research project and subsequently archived in an anonymous format.

The data generated during the trial, at the First Hospital of China Medical University, will be provided at no cost, and the digital object identifier systems for these data will be made available, once articles utilizing the data are published. Issues pertaining to security, licensing, or ethical requirements regarding the intended data are not expected. Any data associated with a previously published article will be included with the current article on publication.

### Plans for collection, laboratory evaluation, and storage of biological specimens for genetic or molecular analysis in this trial/future use {33}

N/A. Our trial does not involve biological specimens for genetic or molecular analysis.

## Statistical methods

### Statistical methods for primary and secondary outcomes {20a}

The oxygenation index data for the two cohorts will be collected, based on a preliminary experiment. The oxygenation index values will be presented as means and standard deviations.

The Student’s *t*-test will be used to assess the disparities between the two cohorts for continuous outcome variables adhering to a normal distribution. In cases of non-normal distribution, the Mann–Whitney test will be used. Categorical data will be analyzed, using the chi-squared or Fisher’s exact tests. These tests are one-sided, and statistical significance will be set at *p* < 0.025. The parameters will be computed and displayed, including approximations and a 97.5% confidence interval.

The primary and secondary outcome indicators will be evaluated using linear and generalized linear mixed-effects models.

A linear mixed-effects model was employed to analyze the primary outcome measures (oxygenation index and quantitative variables). The model included patient baseline data (age, gender, BMI, and prior SARS-CoV-2 infection history), intraoperative characteristics (anesthesia duration, intraoperative crystalloid volume, intraoperative colloid volume, surgical duration, surgical position, pneumoperitoneum pressure, intraoperative PEEP, occurrence of pulmonary re-expansion, intraoperative ventilator tidal volume, and airway platform pressure), and center and group factors.

For quantitative secondary outcome measures—including postoperative PACU oxygen duration, 24-h and 48-h oxygen therapy duration, and postoperative blood gas parameters (pH, partial pressure of carbon dioxide, and lactate) at 48 h—linear mixed-effects models were employed. The model included patient baseline characteristics (age, gender, BMI, and prior SARS-CoV-2 infection history) and intraoperative parameters (anesthesia duration, intraoperative crystalloid volume, colloid volume, surgical duration, surgical position, pneumoperitoneum pressure, intraoperative PEEP, occurrence of pulmonary re-expansion, and ventilator tidal volume), along with center and group factors.

For qualitative data of secondary outcomes—including postoperative PACU oxygenation rate, 24-h postoperative oxygenation rate, 48-h postoperative oxygenation rate, pulmonary complications at 7 days postoperatively, and 30-day mortality rate—generalized linear mixed-effects models were employed. The model included patient baseline data (age, gender, BMI, and history of SARS-CoV-2 infection), intraoperative characteristics (anesthesia duration, intraoperative crystalloid volume, intraoperative colloid volume, surgical duration, surgical position, pneumoperitoneum pressure, intraoperative PEEP, occurrence of pulmonary re-expansion, intraoperative ventilator tidal volume, and airway platform pressure), and center and group factors.

For ordinal outcomes such as postoperative PACU oxygen concentration, 24-h postoperative oxygen concentration, 48-h postoperative oxygen concentration, and severity of pulmonary complications, a generalized linear mixed-effects model was employed. The model included patient baseline data (age, gender, BMI, history of SARS-CoV-2 infection), intraoperative characteristics (anesthesia duration, intraoperative crystalloid volume, intraoperative colloid volume, surgical duration, surgical position, pneumoperitoneum pressure, intraoperative PEEP, occurrence of pulmonary re-expansion, intraoperative ventilator tidal volume, and airway platform pressure), and center and group factors.

### Interim analyses {21b}

The sample size of this study is 1098. Interim analyses are planned to be conducted at three time points: 1 month after the first patient is enrolled, when 50% of the samples are collected, and when 75% of the samples are collected.

### Methods for additional analyses (e.g., subgroup analyses) {20b}

#### Analysis sets

1. The enrolled set will consist of all participants who provided informed consent and were added to the study database.
2. The full analysis set (FAS) describes the analysis set, which is as complete and close as possible to the intention-to-treat ideal of including all randomized participants, who have undergone ≥ 1 efficacy assessment, post-randomization.
3. The per-protocol set (PPS) defines a subset of the participants in the FAS who are more compliant with the protocol and have undergone ≥ 1 efficacy assessment, post-randomization. Participants with protocol deviations will be excluded from this analysis.

### Methods in analysis to handle protocol nonadherence and any statistical methods to handle missing data {20c}

In the analysis of the primary outcome measure, missing values within the FAS will be excluded.

The analysis of the primary, secondary, and safety outcome measures will be conducted, based on the intention-to-treat principle. Thus, participants will be analyzed, according to either of the intervention cohorts to which they were randomized, and all participants will be included in the analysis, regardless of whether they were assigned to the intervention or not (the FAS).

### Plans to give access to the full protocol, participant-level data, and statistical code {31c}

The full protocol, participant-level dataset, and statistical code can be reasonably obtained from the corresponding author.

## Oversight and monitoring

### Composition of the coordinating center and trial steering committee {5d}

The trial will follow the principles of the protocol, GCP, and Declaration of Helsinki. The trial steering committee is composed of the principal investigator and the co-investigator. In addition, the trial is supported by a professional contract research organization (CRO) that provides project management and clinical monitoring services.

### Composition of the data monitoring committee and its role and reporting structure {21a}

The Data Monitoring Committee is composed of experienced clinicians and statisticians. Monitors will ensure participant enrollment, review informed consent forms, and verify source data and CRF entries. Moreover, they will regularly supervise and monitor the trial progress, to support the clinical trial site investigators in adhering to the protocol, in addition to meeting regulatory and ethical requirements. The clinical trial site investigator will report any AEs to the PI and IRB of the First Hospital of China Medical University, as per the standard operating procedures and policies of the local independent ethics committee.

### Adverse event reporting and harms {22}

AEs are defined as any unpredictable or adverse clinical outcome related to any medical intervention that occurred during the study period.

In accordance with the GCP guidelines, any AE or SAE will be documented on the CRF of the participant using an AE/SAE CRF. This will include details, including the type, date, onset, severity, relationship to intervention, management, and outcome of the AE or SAE. Closely monitoring for all AEs and SAEs is essential. Prompt treatment should be provided, if necessary, based on clinical discernment. Follow-up on the AE or SAE should continue until the event has completely resolved and the participant’s condition is stable or normal.

### Frequency and plans for auditing trial conduct {23}

Following feasibility approval by investigators at the initiation meeting, the initial data review will be conducted 30 days post-enrollment of the first subject at each center. Periodic meetings will be convened to assess study progress and address issues at each center. Data surveillance and aggregated adverse event (AE) feedback will be performed upon the enrollment of every 100 subjects.

### Plans for communicating important protocol amendments to relevant parties (e.g., trial participants and ethical committees) {25}

The principal investigator will notify the Trial Steering Committee and disseminate the amended protocol. Following approval via the feasibility assessment, the amendment will be submitted for the clinical trial registry. This filing will include detailed documentation of the modifications and any associated protocol deviations.

## Dissemination plans {31a}

Trial results will be disseminated through publication channel.

## Discussion

This study aimed to explore whether a low FiO_2_ can increase the oxygenation index 48-h post-laparoscopic gastrointestinal surgery and reduce the incidence of PPCs in older adult participants. This study will potentially provide solid evidence for clinical anesthesiologists to choose the most appropriate FiO_2_ for older adult patients undergoing anesthesia, based on lung-protective ventilation strategies. Moreover, the findings can possibly result in the reduction of postoperative adverse events.

A previous study has revealed that a low FiO_2_of 30% did not reduce the incidence of PPCs, postabdominal surgery [45]. The age range of this study population was 40 to 68 years old. Thus, for those > 65 years old, further clinical research is required.

Another article published in 2017 has revealed that a high FiO_2_ is associated with PPCs and an increased 30-day mortality rate in a dose-dependent manner.^46^ The study population included those aged approximately 55 years old, and the study has not been designed for older adults.

Therefore, the effect of oxygen concentration on the postoperative lung status in older adult patients needs to be evidenced by clinical research involving larger study populations.

## Trial status

The planned study start date is 2 July 2024, with an estimated end date of 31 December 31, 2025.

## Supplementary Information

Additional file 1. SPIRIT Checklist for Trials.

## Data Availability

Obtain the consent of the principal investigator have access to the final trial dataset.

## Abbreviations

PPCs: Postoperative pulmonary complications
FiO_2_: Fraction of inspired oxygen
WHO: World Health Organization
ICU: Intensive care unit
PCV: Pressure-controlled ventilation
VCV: Volume-controlled ventilation
BIS: Bispectral index
PEEP: Positive end-expiratory pressure
MAC: Minimum alveolar concentration
AEs: Adverse events
SAEs: Serious adverse events
PACU: Postanesthetic care unit
PI: Principal investigator
CRF: Case report form
eCRF: Electronic CRF
FAS: Full analysis set
PPS: Per-protocol set
CRO: Contract research organization
